# Supplementary figures and images for: Consistent ultra-long DNA sequencing with automated slow pipetting
Source: BMC Genomics. 2021 Mar 12;22:182. doi: 10.1186/s12864-021-07500-w (PMC7953553; doi:10.1186/s12864-021-07500-w)

A

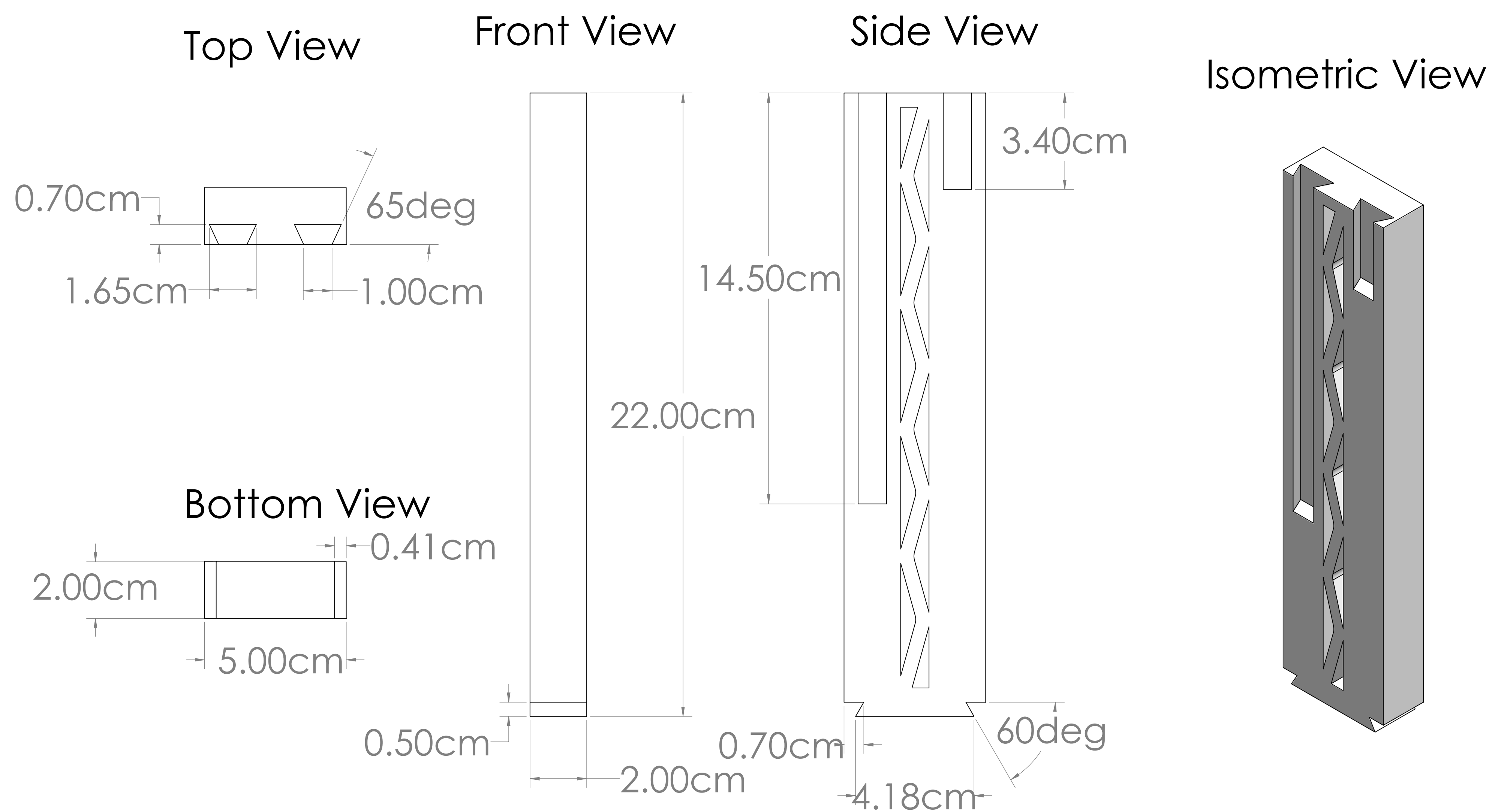

B

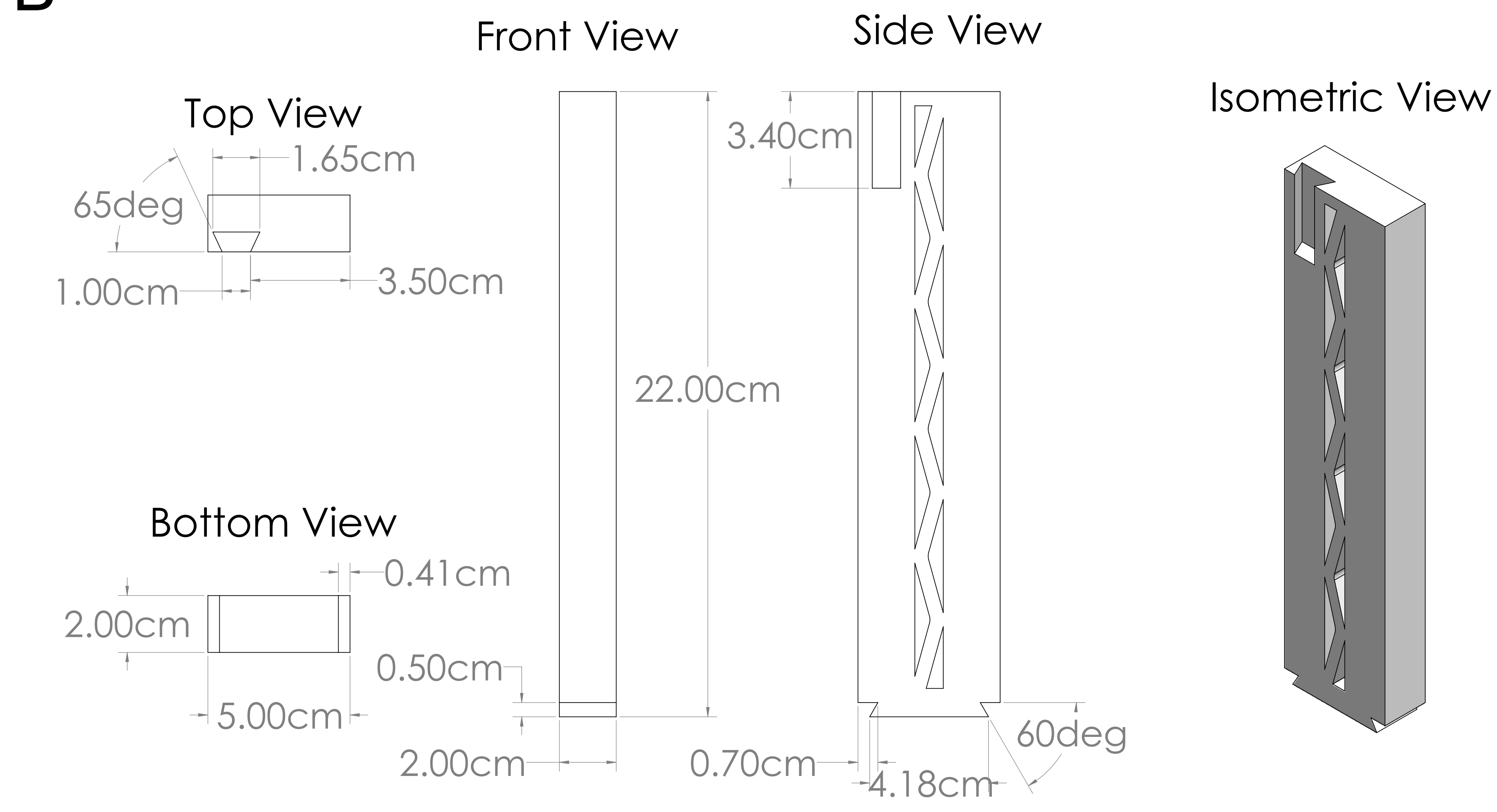

C

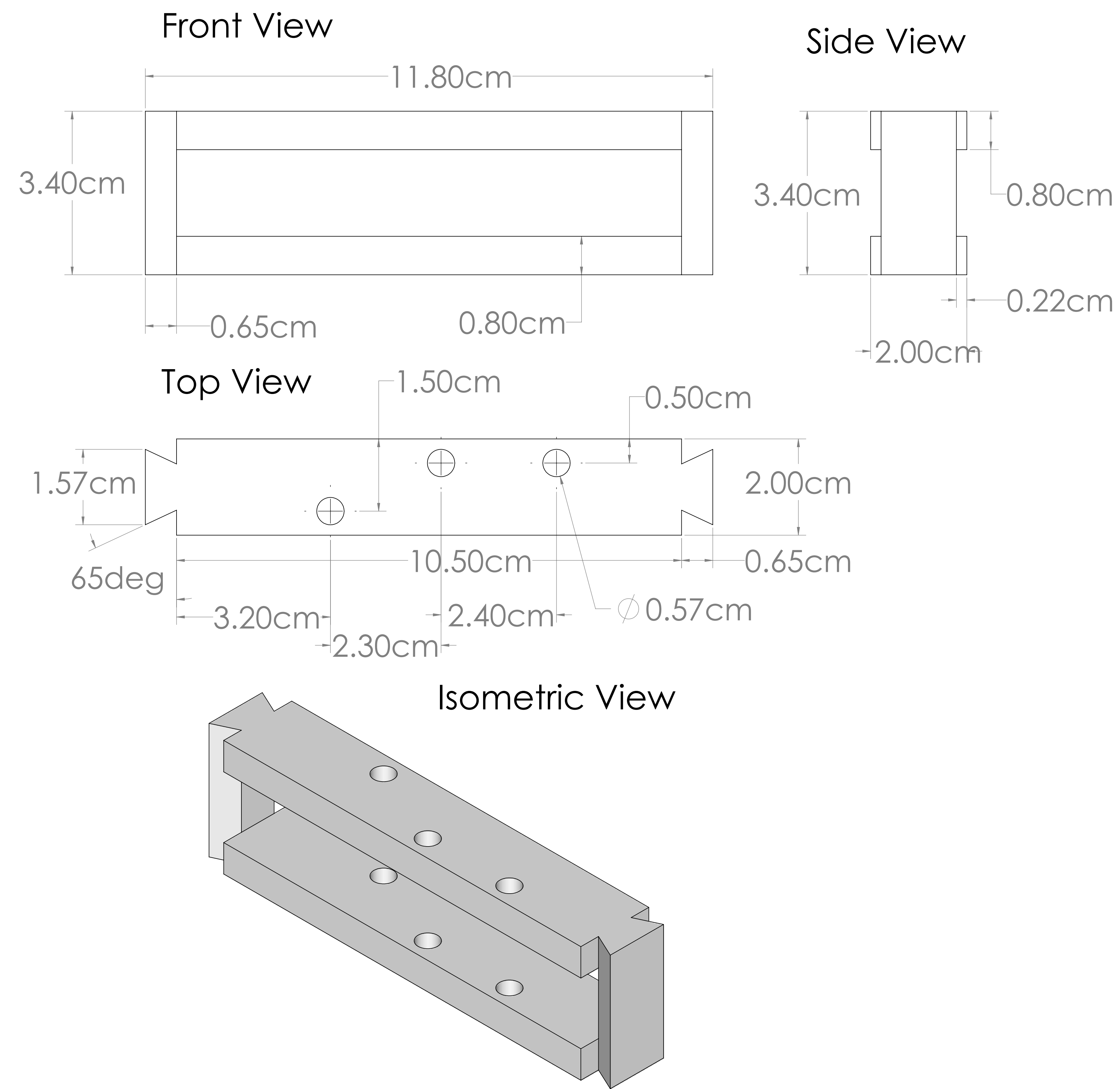

Supplement: Supplementary file 1 — Additional file 1: Supplementary Figure 1. Schematics of SNAILS towers and bridge. A. Dimensions of the right, dovetailed tower. B. Dimensions of the left, dovetailed tower. C. Dimensions of the dovetailed bridge which houses gear axles. [file 12864_2021_7500_MOESM1_ESM.pdf]

A

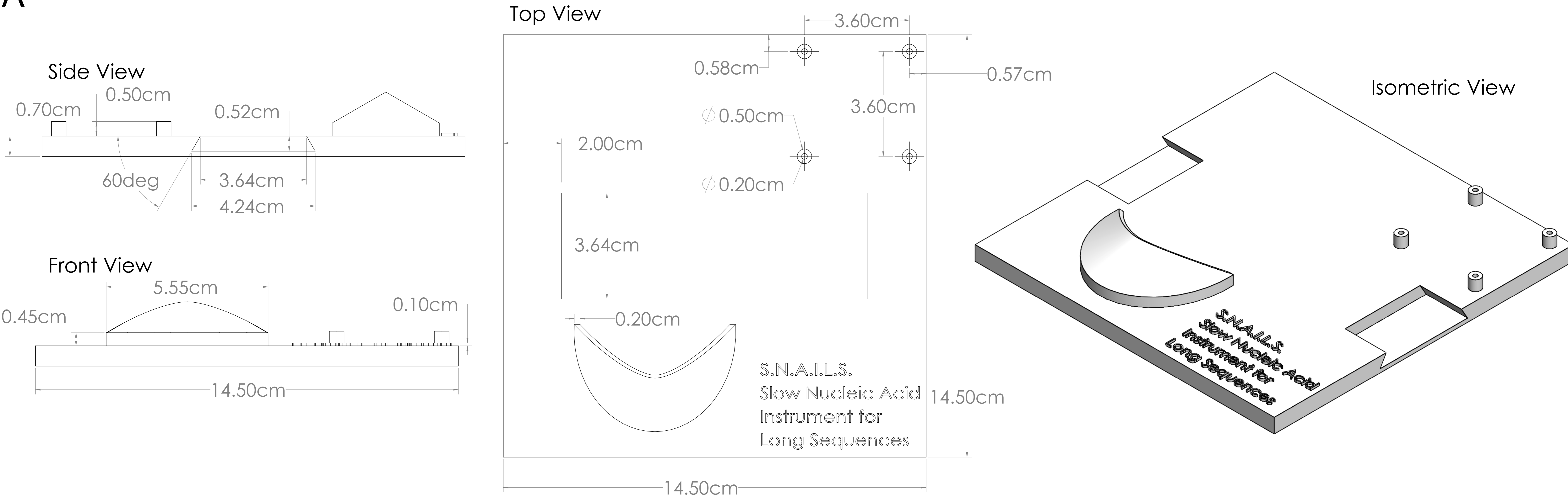

B

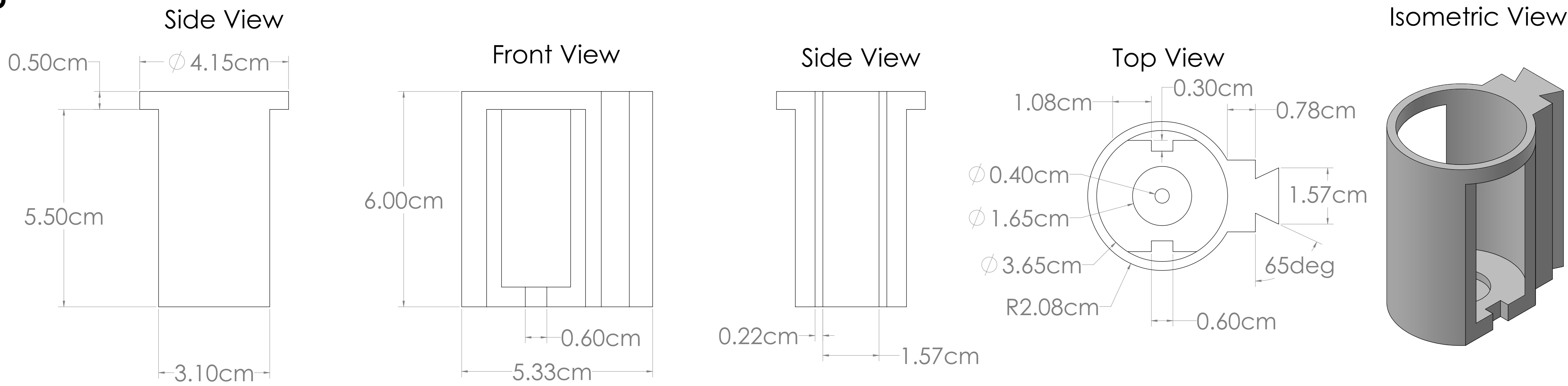

Supplement: Supplementary file 2 — Additional file 2: Supplementary Figure 2. Schematics of SNAILS base and motor basket. A. Dimensions of the base. The slots on either side of the base accept each towers’ dovetail joints. The base is designed with a protruding slot that fits around the base of an Ovation M micropipette. The back right corner includes standoffs aligned to L298N boards for attachment. B. Dimensions of the dovetailed motor basket. The basket is fitted to a 12 rpm HD premium planetary gear motor. [file 12864_2021_7500_MOESM2_ESM.pdf]

**A**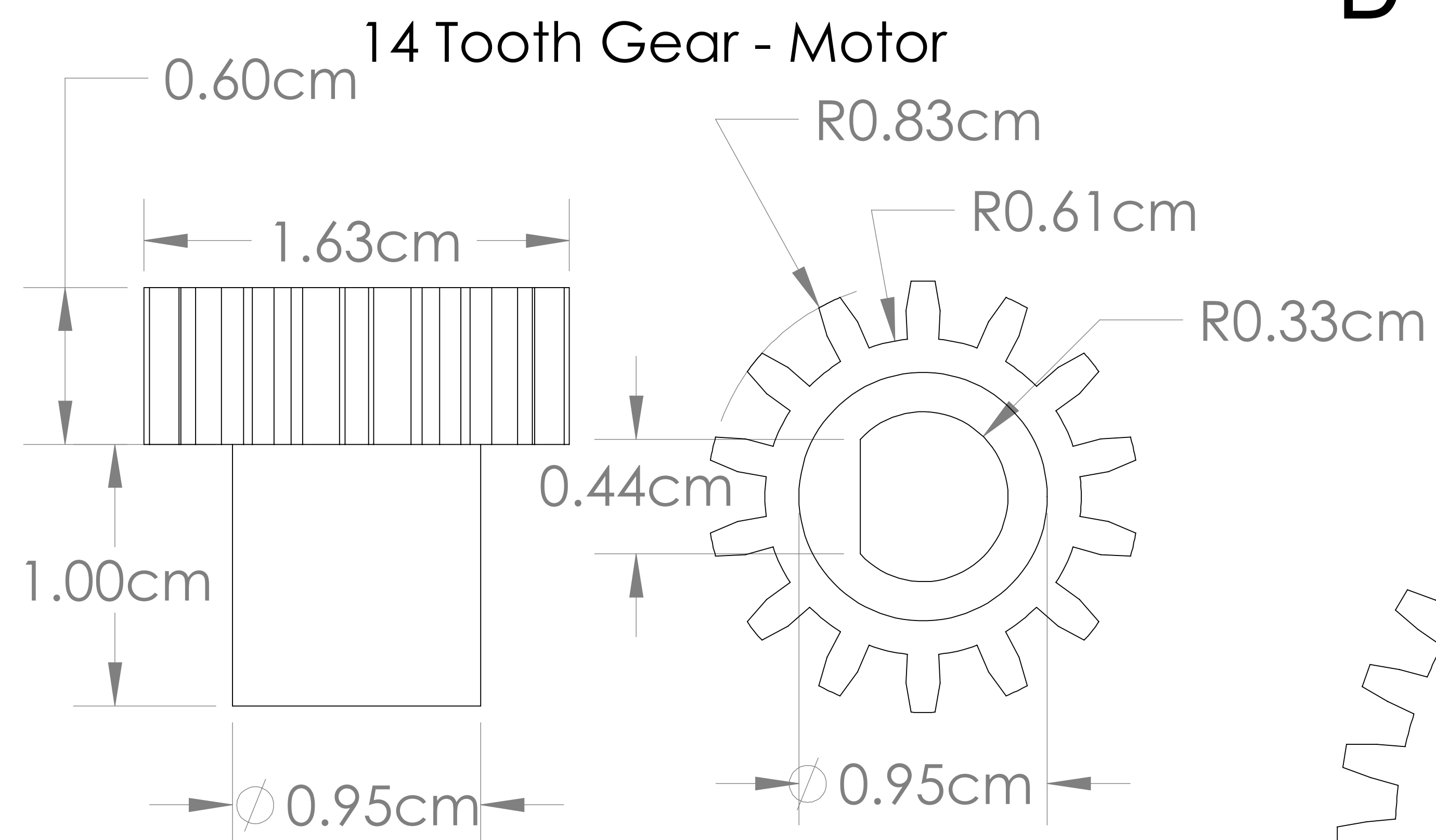**B**

16 Tooth Gear - Axle 2

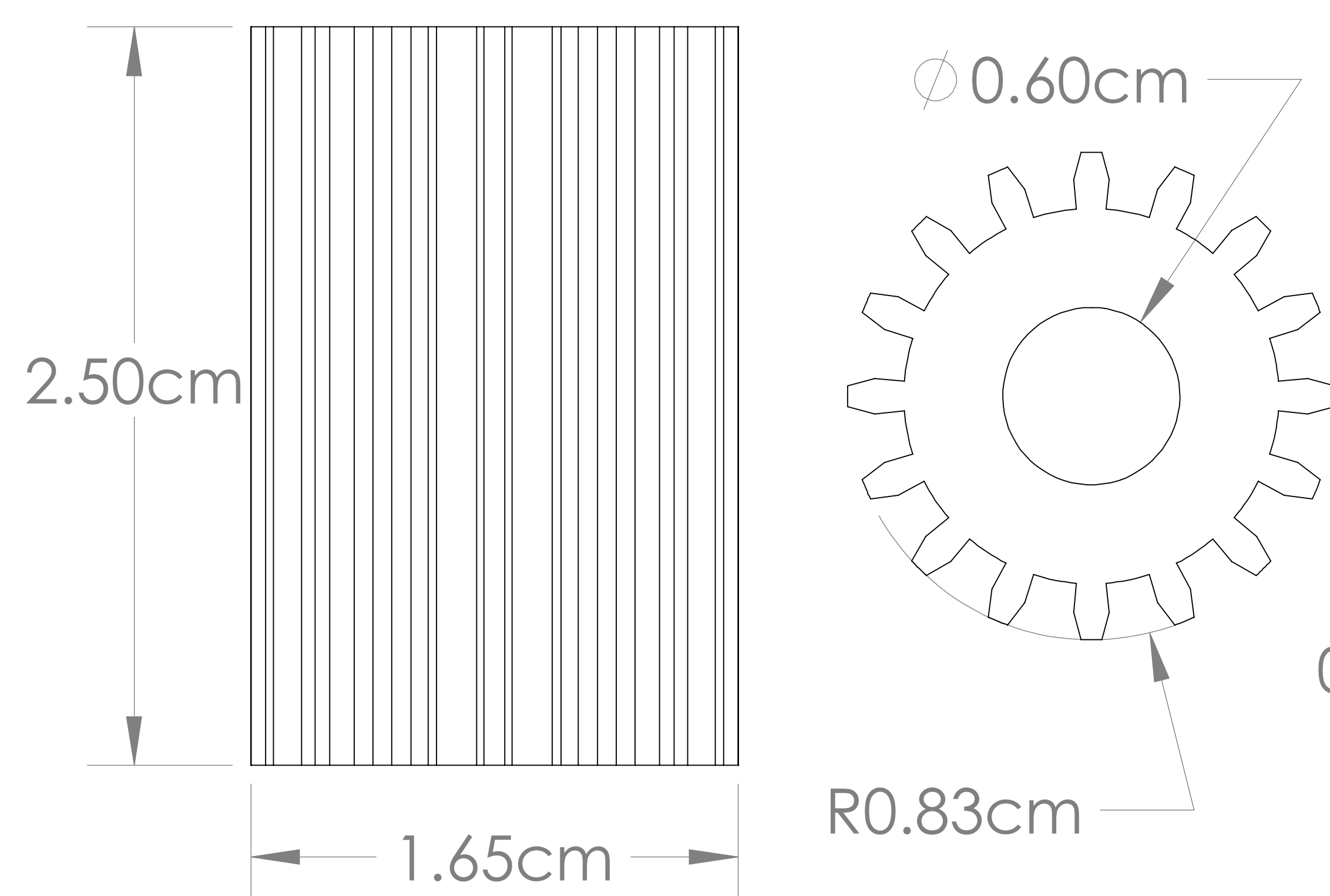**C**

24 Tooth Gear - Axle 2

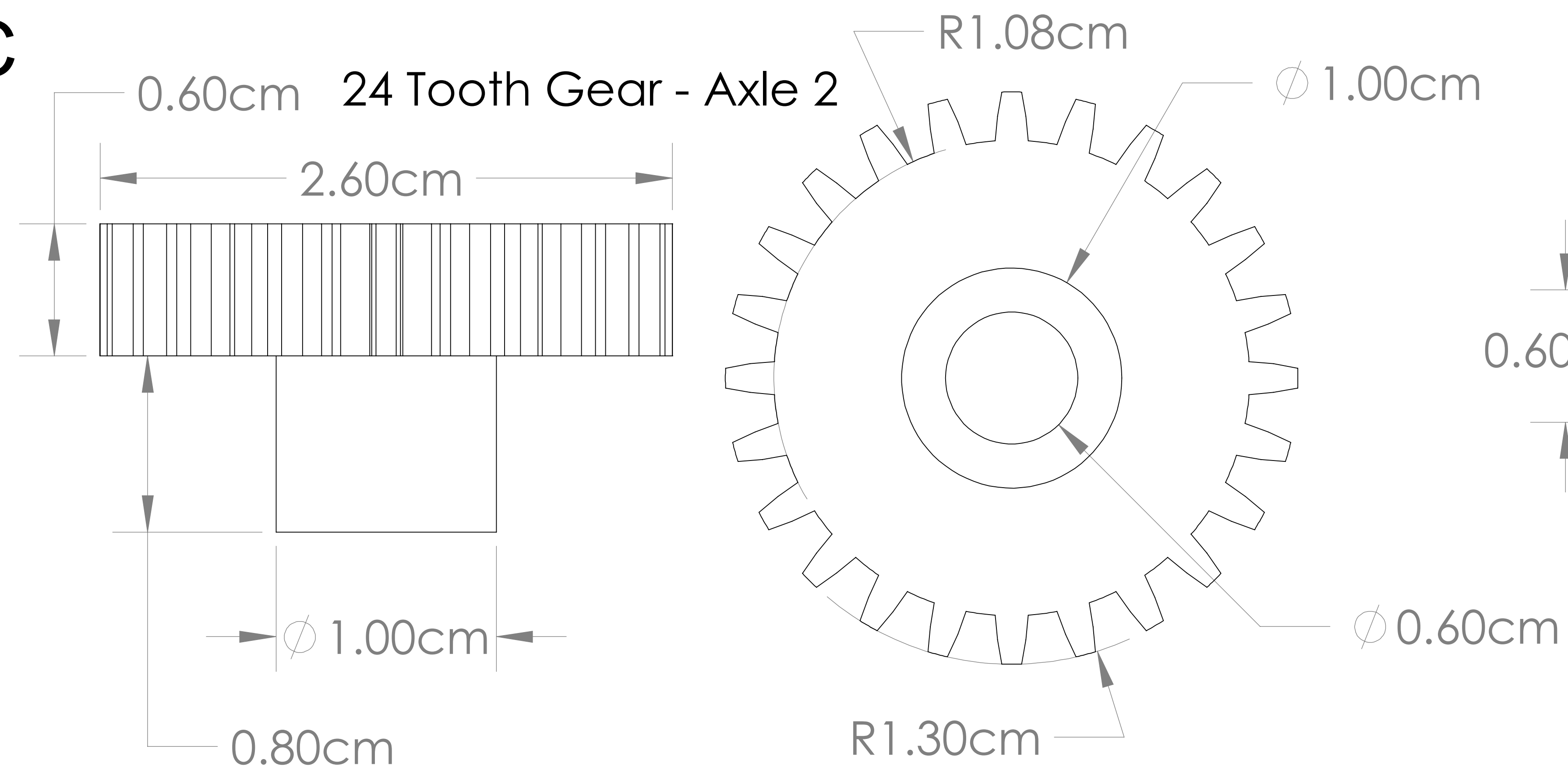**D**

36 Tooth Gear - Axle 1

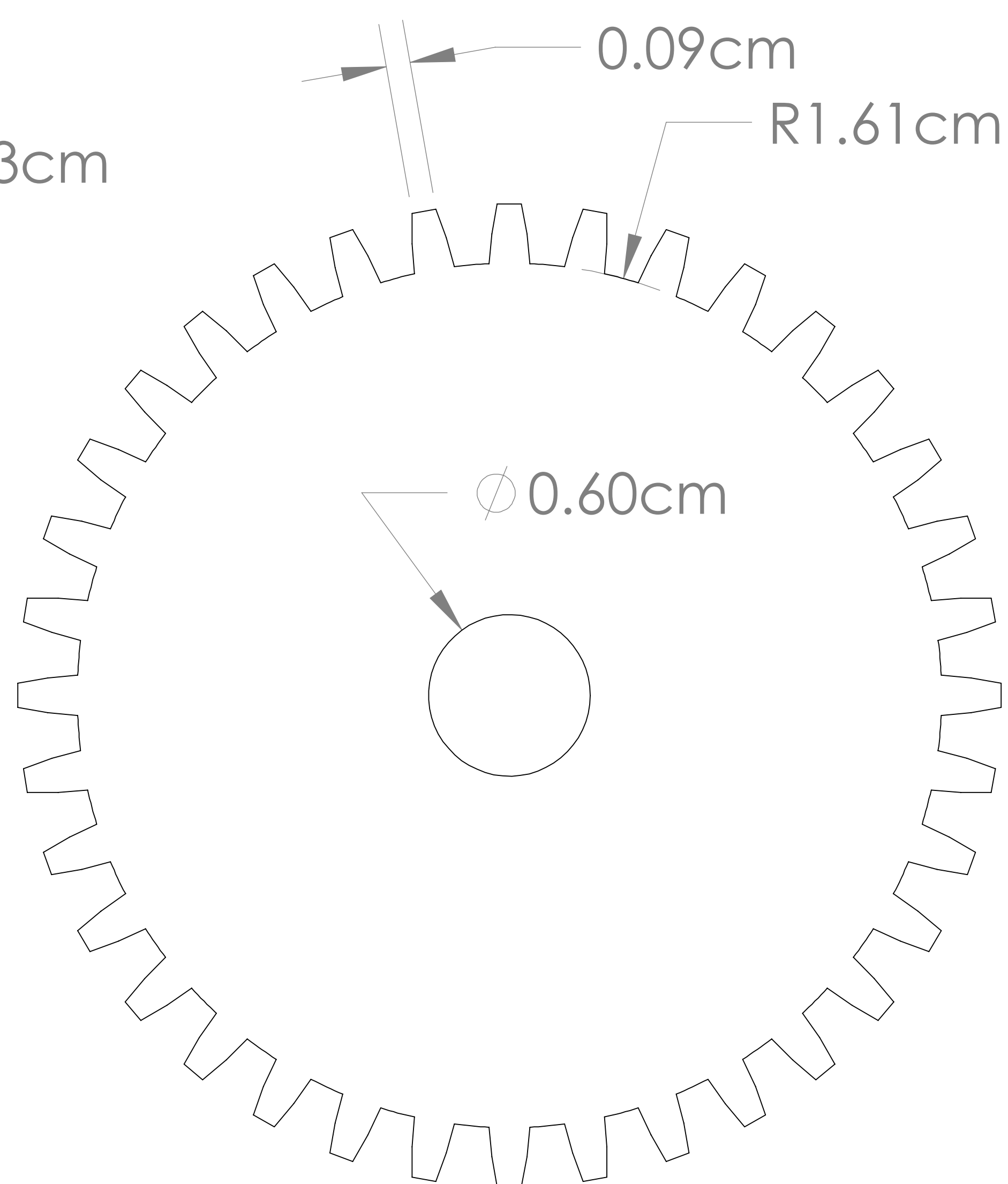**E**

36 Tooth Gear - Axle 3

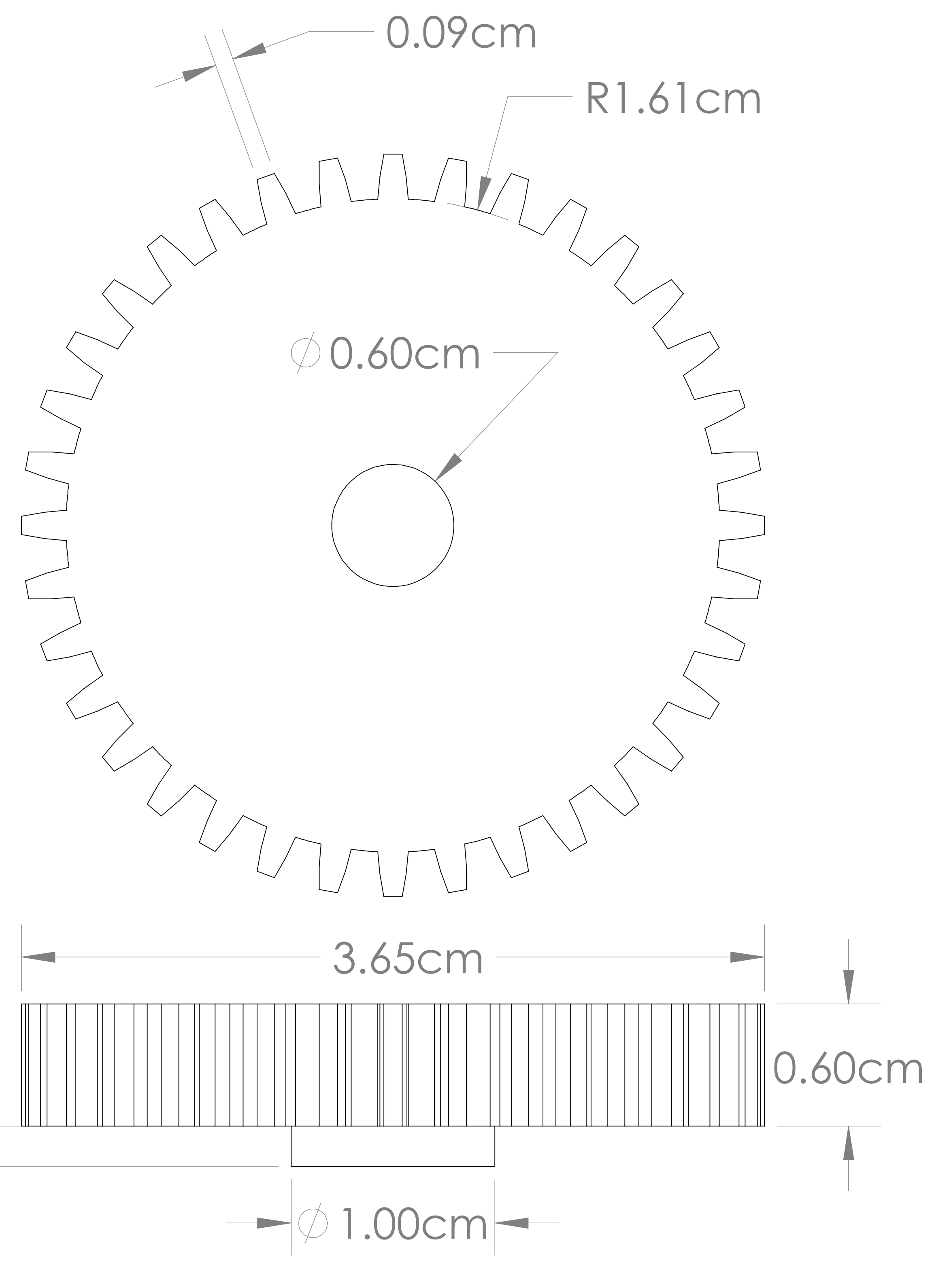**F**

24 Tooth Gear - Axle 3

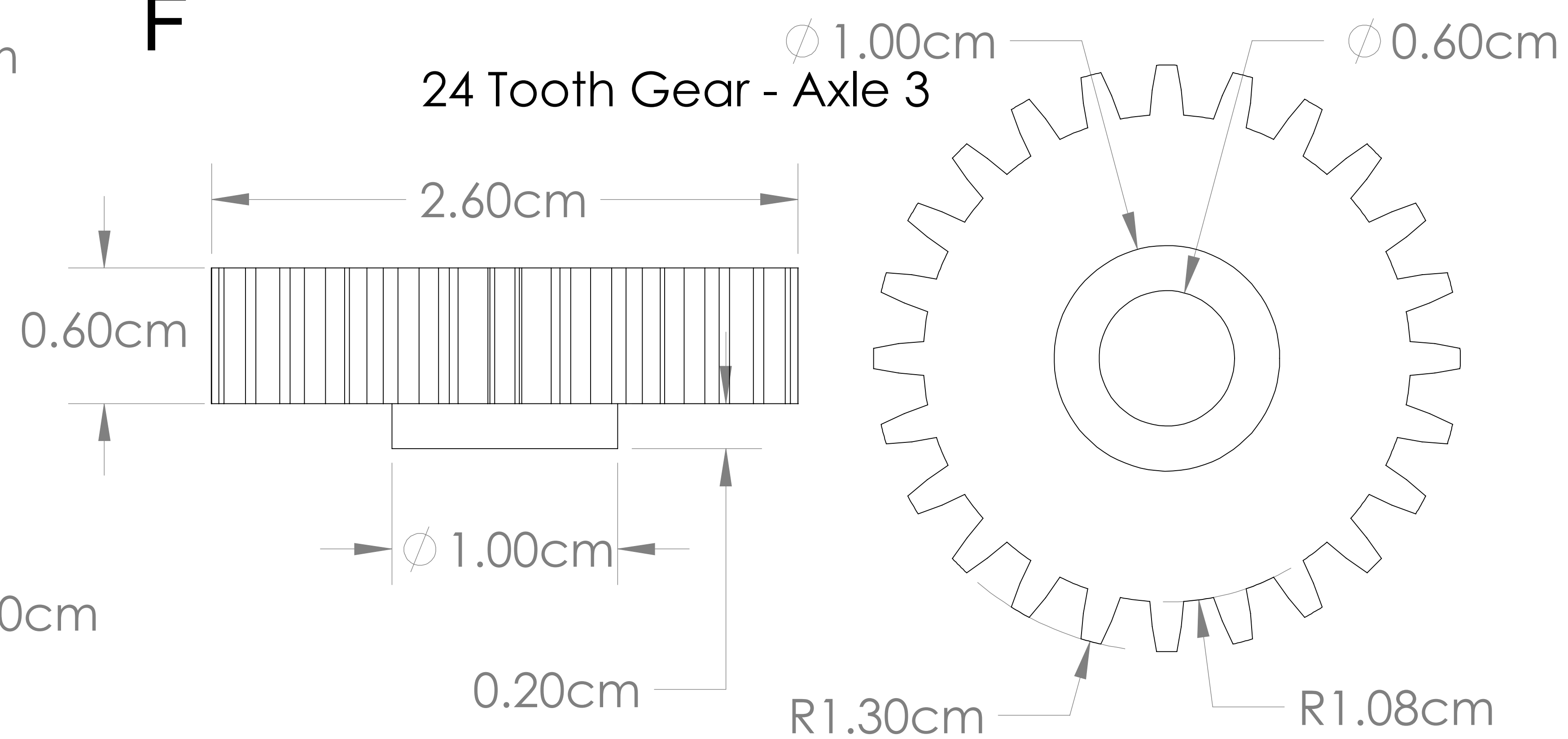

Supplement: Supplementary file 3 — Additional file 3: Supplementary Figure 3. Schematics of SNAILS gears. A. Dimensions of 14 tooth gear cap for planetary motor. B. Dimensions for 16 tooth axle 2 gear. C. Dimensions of 24 tooth axle 2 gear. D. Dimensions of 36 tooth axle 1 gear. E. Dimension of 36 tooth axle 3 gear. F. Dimensions of 24 tooth axle 3 gear. [file 12864_2021_7500_MOESM3_ESM.pdf]

A

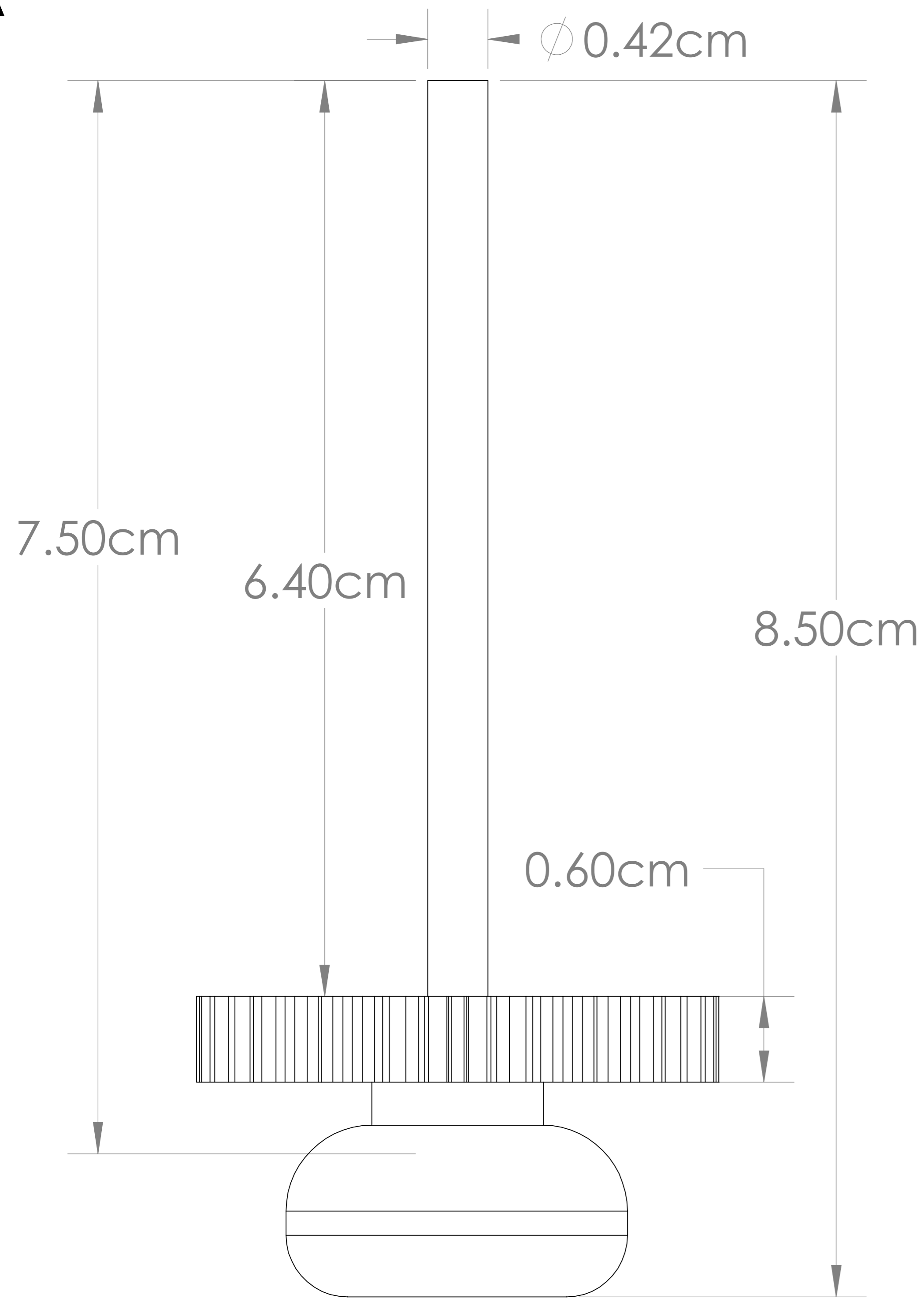

Axle 1 Assembly

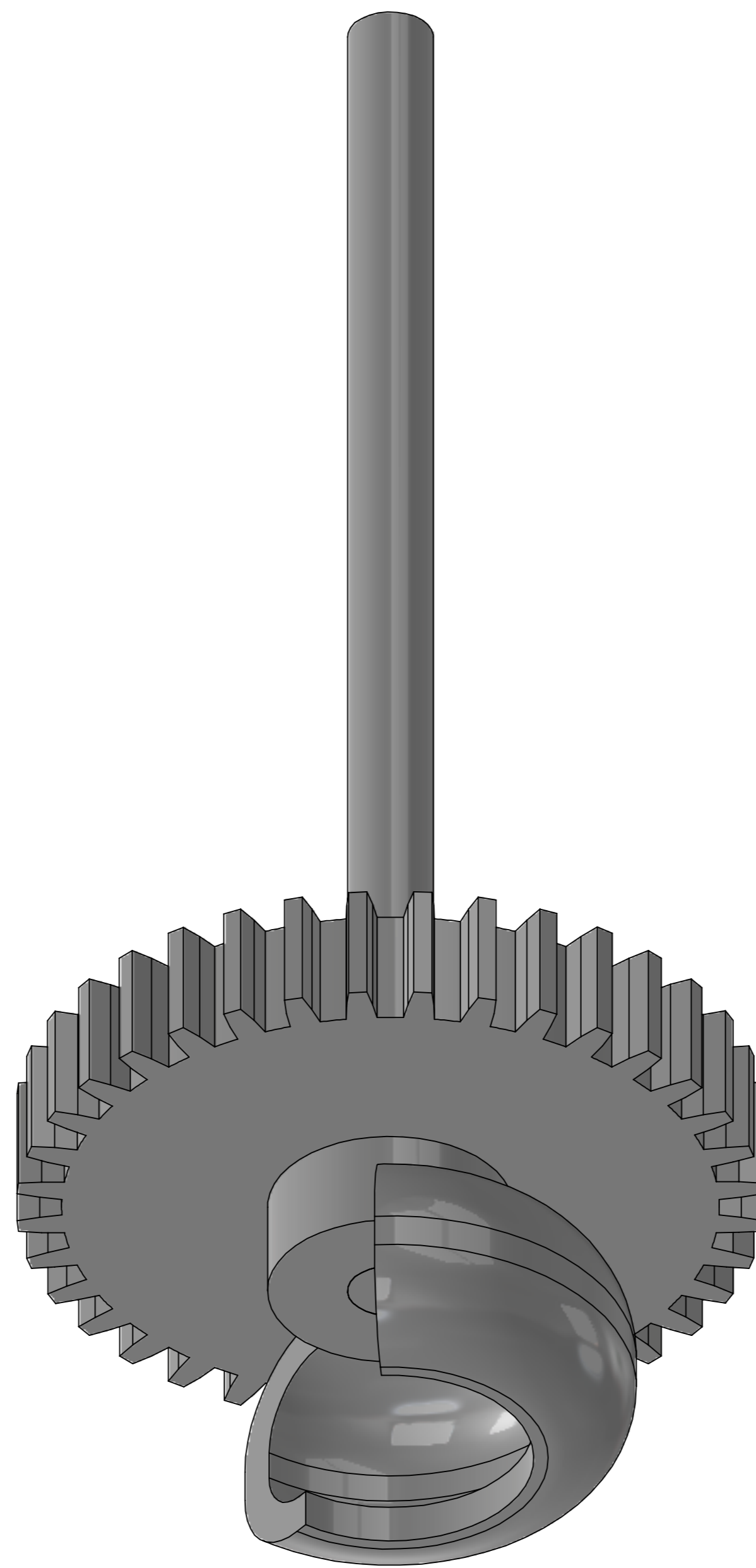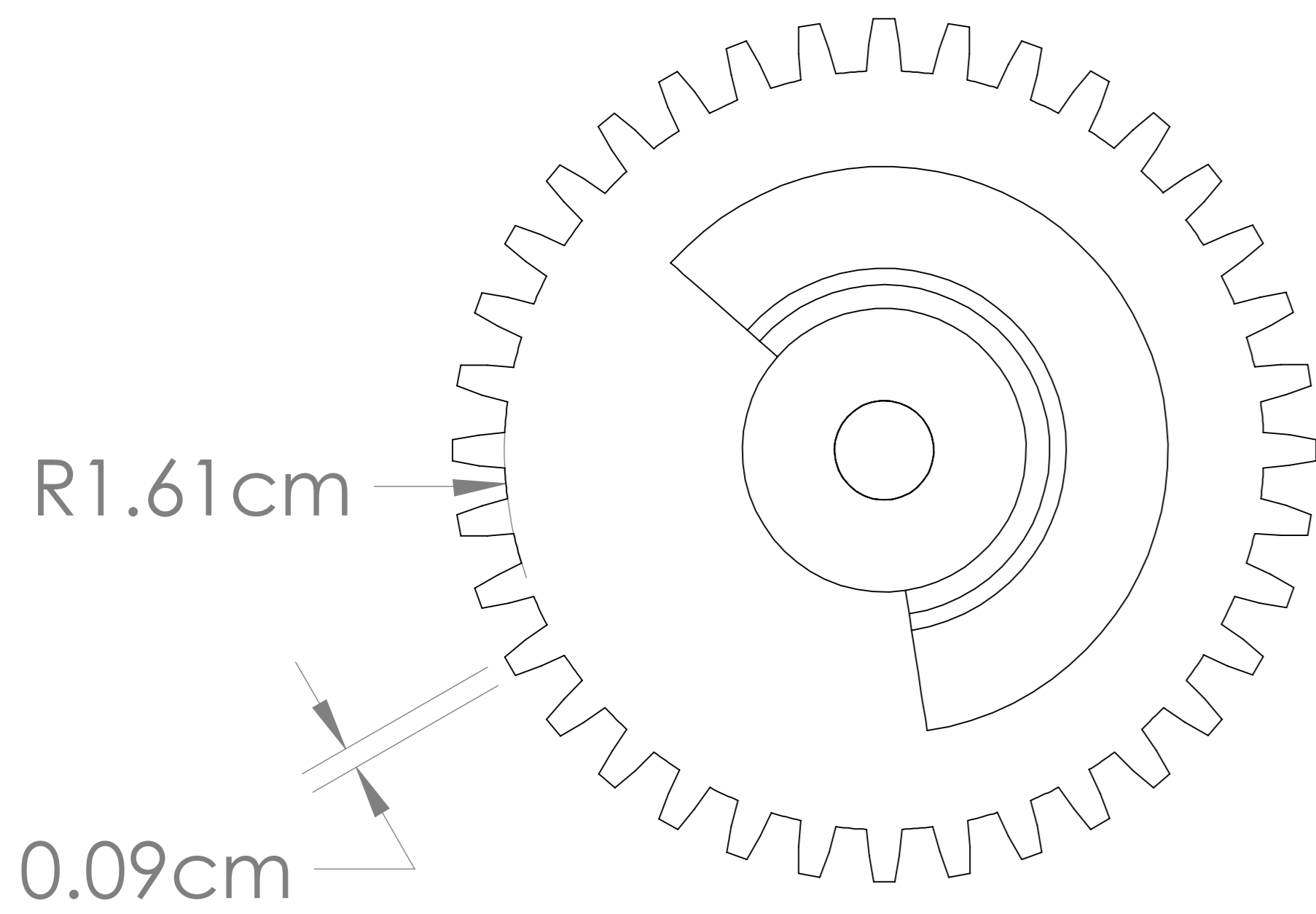

B

Pipette Plunger Cap

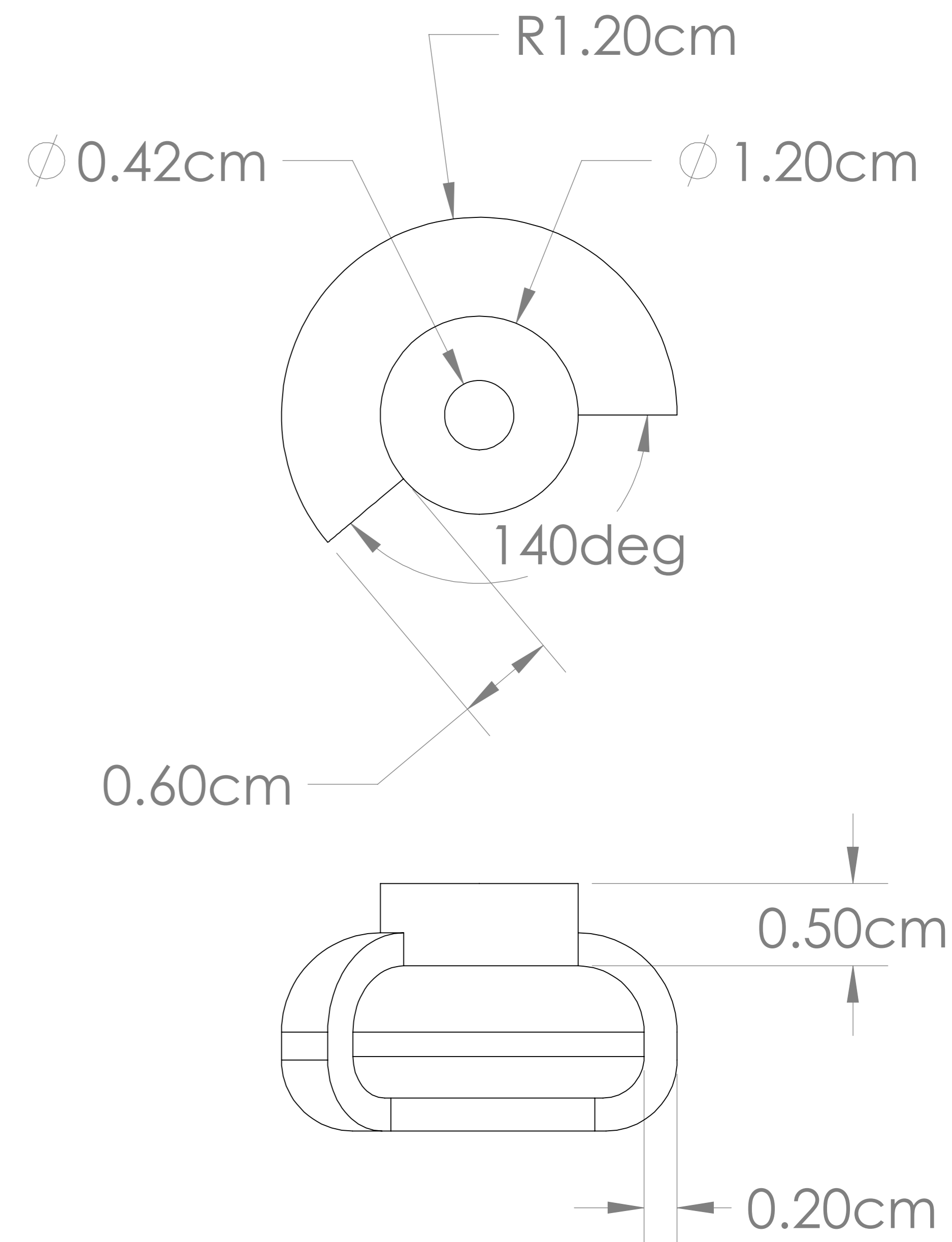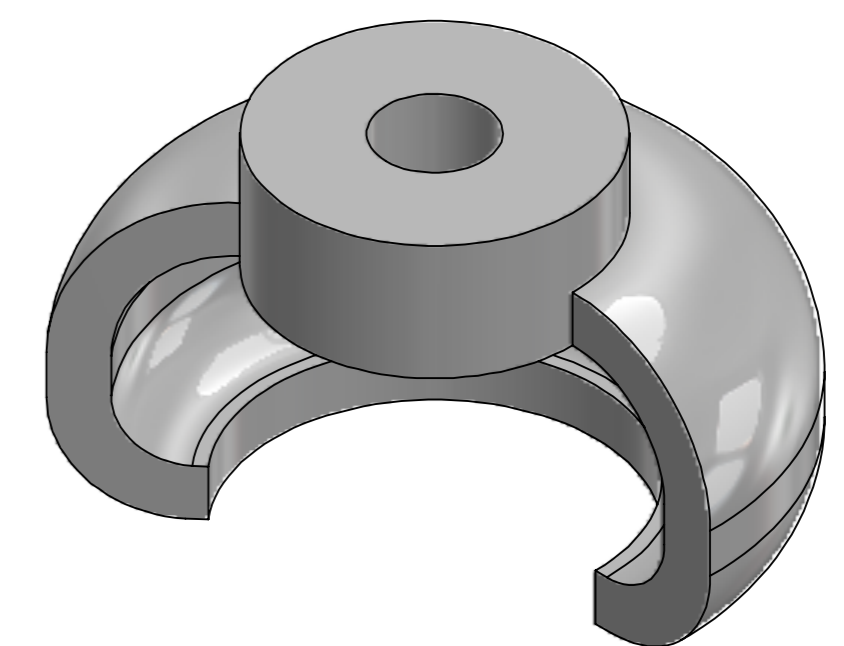

Supplement: Supplementary file 4 — Additional file 4: Supplementary Figure 4. Schematics of SNAILS axle 1 and pipette cap. A. Dimensions for axle 1. B. Dimensions for pipette cap. The cap is fitted for an Ovation M Micropipette. [file 12864_2021_7500_MOESM4_ESM.pdf]
